# Voluntary Medical Male Circumcision for HIV Prevention: New Mathematical Models for Strategic Demand Creation Prioritizing Subpopulations by Age and Geography

**DOI:** 10.1371/journal.pone.0160699

**Published:** 2016-10-26

**Authors:** Catherine Hankins, Mitchell Warren, Emmanuel Njeuhmeli

**Affiliations:** 1 London School of Hygiene and Tropical Medicine, London, United Kingdom; 2 Amsterdam Institute for Global Health and Development, Amsterdam, the Netherlands; 3 AVAC, New York, New York, United States of America; 4 USAID, Washington, District of Columbia, United States of America; University of Pittsburgh, UNITED STATES

## Abstract

Over 11 million voluntary medical male circumcisions (VMMC) have been performed of the projected 20.3 million needed to reach 80% adult male circumcision prevalence in priority sub-Saharan African countries. Striking numbers of adolescent males, outside the 15-49-year-old age target, have been accessing VMMC services. What are the implications of overall progress in scale-up to date? Can mathematical modeling provide further insights on how to efficiently reach the male circumcision coverage levels needed to create and sustain further reductions in HIV incidence to make AIDS no longer a public health threat by 2030? Considering ease of implementation and cultural acceptability, decision makers may also value the estimates that mathematical models can generate of immediacy of impact, cost-effectiveness, and magnitude of impact resulting from different policy choices. This supplement presents the results of mathematical modeling using the Decision Makers’ Program Planning Tool Version 2.0 (DMPPT 2.0), the Actuarial Society of South Africa (ASSA2008) model, and the age structured mathematical (ASM) model. These models are helping countries examine the potential effects on program impact and cost-effectiveness of prioritizing specific subpopulations for VMMC services, for example, by client age, HIV-positive status, risk group, and geographical location. The modeling also examines long-term sustainability strategies, such as adolescent and/or early infant male circumcision, to preserve VMMC coverage gains achieved during rapid scale-up. The 2016–2021 UNAIDS strategy target for VMMC is an additional 27 million VMMC in high HIV-prevalence settings by 2020, as part of access to integrated sexual and reproductive health services for men. To achieve further scale-up, a combination of evidence, analysis, and impact estimates can usefully guide strategic planning and funding of VMMC services and related demand-creation strategies in priority countries. Mid-course corrections now can improve cost-effectiveness and scale to achieve the impact needed to help turn the HIV pandemic on its head within 15 years.

## Introduction

Voluntary medical male circumcision (VMMC) program scale-up for HIV prevention has been under way in settings with generalized HIV epidemics and low prevalence of male circumcision, as recommended in 2007 by the World Health Organization (WHO) and the Joint United Nations Programme on HIV/AIDS (UNAIDS) [1]. More than 11 million males in high HIV-prevalence settings in sub-Saharan Africa have been circumcised [2] of the projected 20.3 million needed to reach 80% adult male-circumcision prevalence in priority countries by the end of 2015 [3]. Undoubtedly, this is substantial progress, but will staying the course with current strategies maximize HIV prevention benefits? Demand for VMMC services has been lower than expected among men 30 years of age and older, while circumcision of young males ages 10–14 years—an age group outside the original target of 15- to 49-year-olds—accounts for nearly 35% of procedures performed to date [4]. Thus, it is timely for decision makers to examine the implications of what has been achieved thus far and to consider prioritization strategies informed by the results of mathematical modeling. Although broader impact would be achieved if simply more men became circumcised, efficient resource utilization would mandate strategic choices about who should be offered VMMC.

## Current Context

In 2015, the UNAIDS/Lancet Commission spotlighted the urgent need to scale up AIDS efforts, get serious about HIV prevention, and continue expanding access to treatment [5]. The United States President’s Emergency Plan for AIDS Relief (PEPFAR) had already called for doing the right things in the right places and at the right times to achieve an AIDS-free generation [6]. UNAIDS published a Fast Track Strategy to end AIDS as a public health threat by 2030 [7]. It set a short-term goal of reducing the number of new HIV infections globally to fewer than 500,000 by 2020 and a longer-term goal of fewer than 200,000 by 2030 [7]. Strategies included strengthening the HIV treatment cascade to achieve 90-90-90, meaning that 90% of people living with HIV will know their HIV status, 90% of people who know their HIV status will be on antiretroviral treatment (ART), and 90% of people on ART will be virally supressed [7]. By 2030, the strategy aims to increase each of these treatment targets to 95%. Achieving 90-90-90 would mean that by 2020, 73% of all people living with HIV would have the viral suppression that brings individual clinical benefit and reduces HIV transmission risk by 96% [8]. The Fast Track Strategy also called for renewed commitment to, sustained funding for, and scaled-up implementation of HIV prevention programs, including VMMC programs in the priority countries in eastern and southern Africa [9]. The UNAIDS World AIDS Day Report [10] and the 2016–2021 UNAIDS strategy [11] have set a target of an additional 27 million men in high HIV-prevalence settings being voluntarily medically circumcised by 2020, as part of access to integrated sexual and reproductive health services for men.

Achieving these aims at country level requires a broad consensus on national goals to end AIDS, target setting, sustained financing, and commitment to implementing effective programs on the ground. In the face of plateauing international funding for HIV[12, 13], decision makers at the country level have to mobilize increased domestic funding for HIV programs and decide where best to allocate resources for the biggest impact on their HIV epidemics. They are cognizant that every new HIV infection means lifelong treatment need. Although HIV incidence may be on the decline in a given country, its decision makers may have data suggesting that more people are acquiring HIV each day than are being placed on treatment [14]. These decision makers will be in a better position to make difficult choices about which strategies to prioritize, which programs to scale up, and where best to invest efforts to achieve national goals if they are equipped with evidence, analysis, and impact estimates to inform effective decision making for HIV prevention.

No one prevention modality on its own can rout a sexually transmitted HIV epidemic. Combination prevention strategies involving biomedical, behavioral, and structural approaches that work synergistically are essential [15]. VMMC, however, can make a unique contribution to combination prevention: it is a single event without ongoing adherence challenges that has lifetime direct benefits for men, along with indirect benefits for women and even for uncircumcised men [16].

## Progress in VMMC Scale-Up and the Potential of Prioritization

Despite the striking achievement in scaling up VMMC, progress has been uneven across sub-Saharan Africa and within countries. Mathematical modeling in 2011 estimated the number of infections that would be averted for what cost and at what cost-savings to achieve 80% male circumcision prevalence by the end of 2015 in high HIV prevalence countries [17]. It underscored the importance of VMMC within the HIV prevention portfolio and led PEPFAR to include VMMC as one of the three key interventions needed to achieve an AIDS Free Generation. It informed the pace and overall scope of country strategies but did not examine the potential effects of prioritization.

**Fig 1:**
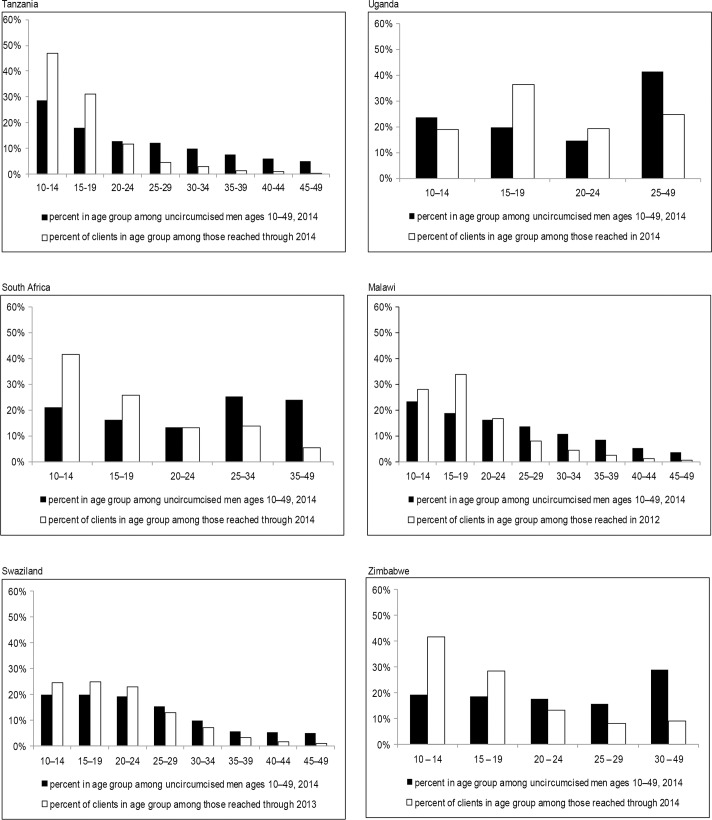
Comparison of VMMC client age groups to their representation in the general male population that is uncircumcised.

In reality, VMMC service scale-up has not necessarily focused on geographic areas with high HIV prevalence within countries, but instead, in many cases, has expanded first in places with greater ease of implementation, as programs focused on achieving an 80% population-level male circumcision prevalence among 15- to 49-year-olds. As well, in many countries, the males coming forward for male circumcision have not always been those at highest risk of acquiring HIV, for whom reductions in HIV incidence might be most immediate. Furthermore, adolescent males have been accessing VMMC services beyond expected numbers in many countries, usually falling outside the lower bounds of age targets initially set by those countries, and disproportionately to their age representation in the general population of uncircumcised males (Fig 1). Although VMMC services should not be denied to any adolescent or adult male who comes forward for male circumcision and who is medically eligible, the funding of VMMC services and related demand creation strategies could prioritize high HIV-incidence settings and certain age groups to achieve bigger impact. However, it is important to realize that adolescent males who are not yet sexually active will naturally age into sexual activity that may put them at risk for HIV, whereas men ages 20–24 will also grow older, entering the potentially higher-risk age categories of 25–29 years and 30–34 years.

What should guide strategic planning and implementation of VMMC programs? Though not the sole information source, mathematical modeling can help to inform decision making by providing insights into potential future scenarios based on today’s programmatic decisions. Decision makers may be most interested in immediacy of impact, or in cost-effectiveness, or in the magnitude of impact of their decisions when they opt for one strategy over another. Alternatively, they may prioritize other factors not addressed by the modeling, such as cultural acceptability and ease of implementation. Ending the HIV epidemic as quickly as possible places priority on immediacy of impact, whereas a longer-term focus values magnitude and sustainability of impact on HIV incidence. Both estimating the number of VMMC procedures that are required to avert one HIV infection and determining the cost-effectiveness of VMMC can allow comparison to other HIV prevention strategies.

If the question is what age group to prioritize for male circumcision, decision makers can compare them. Based on the modeling undertaken for this journal supplement, priority age groups can vary by country and by indicator of interest, as Table 1 shows. Although many countries had initially aimed to achieve 80% male-circumcision prevalence in the age group 15–49 years [3], several countries that participated in the modeling exercises—including Malawi [18], Swaziland [19], Uganda [20], and Zambia [21]—have revised their strategies and/or operational plans to focus on achieving targets among younger age ranges (most frequently 10–34 years) to maximize a combination of impact, cost-effectiveness, and feasibility.

**Table 1:** Priority country-specific age groups by indicator of interest.

Indicator	Malawi [18]	Tanzania [22]	Swaziland [19]	South Africa [23]	Uganda [20]	Zambia [21]	Zimbabwe [24]
Number of VMMC/HIV infections averted	20–34	20–34	15–34	20–34	20–34	15–29	15–34
Immediacy of impact	20–34	20–34	20–29	20–34	20–34	15–24	15–24
Magnitude of impact	15–24	10–24	15–29	15–24	10–19	10–24	10–24
Cost-effectiveness	15–34	15–34	15–34	15–34	15–34	15–29	15–34
PEPFAR target [25, 26]	15–29	10–29	15–29	15–34	15–29	15–29	15–29
New country age target	10–34	10–34	10–34*	TBD	10–34	TBD	13–29

* Swaziland new country age target: 50% coverage for neonates, 80% coverage among males ages 10–29 years, and 50% coverage among males ages 30–34 years.

## New Mathematical Models for Strategic Demand Creation Prioritizing Subpopulations by Age and Geography

This supplement presents new mathematical models that can help countries examine the potential effects of focusing on specific subpopulations for male circumcision services by age and geography. The Decision Makers’ Program Planning Tool, Version 2.0 (DMPPT 2.0), developed by the USAID- and PEPFAR-funded Health Policy Project [27], is a compartmental model that allows the user to analyze the effects of VMMC client age and of geography on program impact and cost-effectiveness. This model was applied to five countries [28], all of which subsequently made policy decisions based on the modeling results.

Based on the DMPPT 2.0 age analysis and the country’s programmatic experience, Malawi’s VMMC strategy now focuses on males ages 10–34 years and prioritizes 14 of the 28 districts in the country [18]. The DMPPT 2.0 model’s results in Tanzania reinforced the VMMC strategy launched in 2010, giving the country new confidence in investing in circumcising adolescents [22]. Modeling helped Swaziland set age-specific targets, balancing cost, impact, and feasibility, that were harmonized with its national goal of 70% coverage by 2018 of men ages 10–49 years [19]. Application of DMPPT 2.0 in Uganda led policymakers to propose males ages 10–34 years as a priority group for VMMC in Uganda’s application to the Global Fund [20]. In South Africa, the DMPPT 2.0 modeling results did not support the geographic prioritization of specific provinces but did reveal that a strategy focusing on men ages 15–34 years would maximize program benefits [23].

An age-structured mathematical (ASM) model developed by researchers at Weil Cornell Medical College, supported by the Bill & Melinda Gates Foundation, was applied to Zambia to determine how subpopulation prioritization could increase program efficiency. It revealed that epidemic impact and program efficiency would be improved by prioritizing young males who are sexually active or just before sexual debut, high HIV-prevalence geographic areas, and men who are at highest risk of HIV acquisition [21]. Likewise, the ASM model applied to Zimbabwe projected that program efficiency would improve by prioritizing young sexually active males and those whose sexual behaviour puts them at higher risk for acquiring HIV [24]. Including males regardless of their HIV status in VMMC programs would increase program effectiveness if it increases VMMC uptake among men at higher risk of HIV acquisition and if VMMC in HIV-positive men reduces their risk of the ulcerative sexually transmitted infections that increase HIV shedding and transmission to women. Importantly, VMMC services can serve as a valuable portal of entry linking men who are tested and found HIV-positive to the antiretroviral treatment that can result in viral suppression and reduced infectivity.

The World Bank supported the adaption of a demographic and epidemiological model of the HIV epidemic in South Africa—the ASSA2008 model—to analyze the impact of VMMC and track resulting financial savings from reduced HIV incidence using a costing module [29]. The return on investment was highest if males are circumcised between the ages of 20 and 25 years, but this return on investment declined with age steeply thereafter. In addition, a costing exercise conducted by the Health Policy Project and the USAID- and PEPFAR-funded Project SOAR (Supporting Operational AIDS Research) that systematically collected cost data from 33 national government- and PEPFAR-supported urban, rural, and peri-urban VMMC facilities in eight of South Africa’s nine provinces [30] revealed that unit costs were significantly higher in public hospitals than in health centers and clinics. Direct costs could be reduced by 17% if South Africa encouraged task shifting from doctors to nurses.

The VMMC modeling collection also includes four papers that explore the impact of scale-up progress thus far [4], the potential cost-effectiveness of increasing demand among 20- to 29-year-olds in Zimbabwe [31] the projected contribution of VMMC scale-up to the Fast Track goals in light of the 90-90-90 and 95-95-95 treatment cascade targets [32], and the cost and impact of introducing early infant male circumcision (EIMC) for long-term sustainability of VMMC programs [33].

The age-specific impact, cost-effectiveness, and coverage attributable to male circumcisions conducted through the end of 2014 were modeled to assess actual progress in comparison with the scenario of achieving 80% coverage among men ages 15–49 years by 2015 in the original 12 priority countries and in Nyanza Province, Kenya [4]. This work used DMPPT 2.1, which is an updated version of DMPPT 2.0 that begins with the onset of each country’s VMMC program and can vary start and end dates for model outputs. The median estimated cost per HIV infection averted based on actual VMMCs performed through 2014 was US$4,400. Strikingly, more than half of the projected HIV infections averted were attributable to circumcising 10- to 19-year-old males.

The Goals module of the Spectrum suite of models [34] was used to assess the impact and cost of VMMC scale-up in the context of the 90-90-90 targets for 2020 and the 95-95-95 targets for 2030 [32]. Several scenarios were examined using data and treatment cascade aspirations for Lesotho, Malawi, South Africa, and Uganda. Across all four countries, scaling up VMMC would provide HIV incidence reductions additional to those achieved by reaching the viral suppression targets of 90-90-90. Once VMMC coverage targets are met, annual program costs (ART and VMMC combined) are lower than in the scenarios in which VMMC coverage remains at 2015 levels. In addition, in the two scenarios in which the 90-90-90 targets were not met, scaling up VMMC reduced HIV incidence to nearly the same levels as scaling up ART to 90-90-90 without further scale-up of VMMC.

The manuscript on progress toward VMMC targets revealed that some countries are reaching 80% coverage among some age groups faster than was initially apparent by focusing on numerical targets alone, raising the possibility that these countries could start planning for sustainability earlier than they expected. What is the best implementation model for long-term sustainability of VMMC programs, so that the gains in coverage achieved during the rapid scale-up are not lost? One alternative that countries can consider is that of routinely offering VMMC to all males in the 10- to 14-year-olds age group, to create a cohort-based replenishment effect. Another alternative is to introduce EIMC strategies. EIMC is a simpler procedure that requires no sutures and that, with parental consent, can be safely performed by trained, experienced, lower-cadre healthcare providers [35]. Although the introduction of EIMC now would increase the overall numbers of circumcisions that are needed over the next decade, models project that this would not increase the long-term costs, assuming that EIMC unit costs are substantially lower than those of adolescent VMMC [33]. A third strategy would be to provide a routine offer of VMMC both for infant males and for 10- to 14-year-olds during the sustainability phase of implementation.

## Conclusion

Progress to date on VMMC scale-up for HIV prevention in sub-Saharan Africa has been truly impressive. Millions of men and adolescents have come forward to receive VMMC in settings with high HIV prevalence and low male-circumcision prevalence. At issue now is how to efficiently reach the levels of male circumcision coverage needed to create and sustain further reductions in HIV incidence toward the 2030 goal of a world in which AIDS is no longer a public health threat.

Modeling work using DMPPT 1.0 (the predecessor of DMPPT 2.0) informed the initial targets of the UNAIDS Joint Strategic Action Framework to Accelerate the Scale-Up of Voluntary Medical Male Circumcision for HIV Prevention in Eastern and Southern Africa 2012–2016 [36]. Likewise, the results of the modeling work using DMPPT 2.0 published in this supplement are expected to play an important role in the development of WHO’s new action framework on VMMC in the context of Fast Track and combination prevention. Moreover, this work can guide country programs as they establish and work toward their new age-specific coverage targets and prepare for sustainability.

The focus is shifting from the original 80% male-circumcision prevalence target for males ages 15–49 years. Countries are being re-energized in their efforts to achieve more feasible, realistic targets that will have nearly the same epidemiologic impact as earlier, more ambitious targets promised. Geographic and age prioritization, combined with tailored, effective demand creation and expanding VMMC choices through the introduction of new devices alongside current conventional surgical techniques [37], will speed the HIV incidence reductions that VMMC can accomplish. It is time now for the mid-course corrections that will improve cost-effectiveness and achieve impact to help turn the HIV pandemic on its head within 15 years.
