# Correction: Voluntary Medical Male Circumcision for HIV Prevention: New Mathematical Models for Strategic Demand Creation Prioritizing Subpopulations by Age and Geography

**DOI:** 10.1371/journal.pone.0169499

**Published:** 2016-12-30

**Authors:** Catherine Hankins, Mitchell Warren, Emmanuel Njeuhmeli

There is an error in the Funding statement. The number for the Cooperative Agreement Project SOAR (Supporting Operational AIDS Research) is incorrect. The correct number is OAA-A-14-00060.
